# Effect of Strain Rate and Silica Filler Content on the Compressive Behavior of RTM6 Epoxy-Based Nanocomposites

**DOI:** 10.3390/polym13213735

**Published:** 2021-10-28

**Authors:** Ahmed Elmahdy, Aldobenedetto Zotti, Simona Zuppolini, Mauro Zarrelli, Anna Borriello, Patricia Verleysen

**Affiliations:** 1 Materials Science and Technology-DyMaLab Research Group, Department of Electromechanical Systems and Metals Engineering, Faculty of Engineering and Architecture, Ghent University, Tech Lane Ghent Science Park, Technologiepark 46, 9052 Zwijnaarde, Belgium; patricia.verleysen@ugent.be; 2 Institute of Polymers, Composites and Biomaterials, National Research Council of Italy, P.Ie Fermi, 1, 80055 Naples, Portici, Italy; aldobenedetto.zotti@unina.it (A.Z.); simona.zuppolini@cnr.it (S.Z.); borriell@unina.it (A.B.)

**Keywords:** epoxy resin, nanocomposites, silica nanoparticles, mechanical behavior, high strain rate, split Hopkinson bar

## Abstract

The aim of this paper is to investigate the effect of strain rate and filler content on the compressive behavior of the aeronautical grade RTM6 epoxy-based nanocomposites. Silica nanoparticles with different sizes, weight concentrations and surface functionalization were used as fillers. Dynamic mechanical analysis was used to study the glass transition temperature and storage modulus of the nanocomposites. Using quasi-static and split Hopkinson bar tests, strain rates of 0.001 s^−1^ to 1100 s^−1^ were imposed. Sample deformation was measured using stereo digital image correlation techniques. Results showed a significant increase in the compressive strength with increasing strain rate. The elastic modulus and Poisson’s ratio showed strain rate independency. The addition of silica nanoparticles marginally increased the glass transition temperature of the resin, and improved its storage and elastic moduli and peak yield strength for all filler concentrations. Increasing the weight percentage of the filler slightly improved the peak yield strength. Moreover, the filler’s size and surface functionalization did not affect the resin’s compressive behavior at different strain rates.

## 1. Introduction

Epoxy resins are widely used as matrix material for high-performance composites in aeronautical applications. They are generally characterized by a high cross-linking density compared to other thermoset polymers. This gives epoxy resins and their composites many advantages such as high stiffness, good chemical resistance, good performance at high temperatures and excellent fatigue performance [1]. Additionally, their low curing shrinkage does not cause curing cracks in large aerospace components. However, because of the high cross-linking density, epoxy resins are generally very brittle with a very low fracture strain and have poor resistance to impact and crack propagation [2]. For this reason, efforts were made to improve the mechanical performance of the epoxy resins by the addition of different types of fillers, such as inorganic particles [3,4,5], elastomer particles [6,7], carbon nanotubes [8,9], hyperbranched polymers [10,11,12] and recently graphene nanoplatelets [2,13]. Compared to other filler types, silica nanoparticles are widely studied as fillers to epoxy resins. This is related to the marginal effect of the silica-based fillers on the glass transition temperature of the hosting epoxy matrix, and hence its curing temperature [14,15,16,17]. Moreover, the advancement in synthesis processes, particularly sol–gel and modified sol–gel techniques, allow the production of these nanoparticles either as precipitates or directly in the epoxy resin itself (in situ) [18], which can be considered for large scale manufacturing of epoxy nanocomposites with a relatively low cost [19]. In addition, these synthesis techniques allow a very high degree of control over the size and distribution of the formed nanoparticles [6]. The addition of silica nanoparticles up to a weight content of 25% generally improves the overall mechanical performance of epoxy resins such as tensile strength and stiffness [15,20], fracture toughness, compressive strength [21] and fatigue crack growth [22]. Additionally, when combined with carbon fibers, the silica nanoparticles can improve the overall toughness of the carbon epoxy composites by enhancing the interfacial adhesion with the fibers [23].

The extent of the improvement of the physical and the mechanical properties of epoxy nanocomposites is highly affected by the size and surface condition of the silica nanoparticles. Surface functionalization of the silica nanoparticles generally improves the compatibility of the particles with the hosting matrix [24] and improves the overall mechanical performance and glass transition temperature of the hosting epoxy resin up to silica particle sizes of 400 nm [25,26,27,28]. With regard to the size, the addition of silica nanoparticles of size ranging from 7 nm to 80 nm does not significantly affect glass transition temperature or the mechanical properties of the hosting epoxy resin [16,25,29]. However, at silica particle size of 100 nm or larger, no clear trends can be established. On the one hand, Dittanet et al. [29] showed that for a silica particle size range of 23 to 170 nm and up to 30% weight content, the mechanical properties and the glass transition temperature of the epoxy remained nearly constant regardless of the silica particle size. On the other hand, Bondioli et al. [30] showed that the elastic modulus of the epoxy resin increased by the addition of 1% weight content of 75 nm silica nanoparticle compared to 330 nm silica nanoparticles, which partially contradicts with the findings of Dittanet et al. [29]. Sun et al. [31] also reported a decrease in the glass transition temperature for epoxy filled with 100 nm silica nanoparticles and 10% weight content, compared to a constant glass transition temperature for the same epoxy filled with 3 µm silica particles at the same weight content, which also contradicts with the findings of Dittanet et al. [29].

As mentioned earlier, the main aim of improving epoxy resins is to enhance their use as matrix material and to improve the performance of aeronautical composite structures. These structures are typically subjected to extreme impact events, such as bird strike or fan blade-out events. Therefore, studying the effect of strain rate on the mechanical behavior of silica/epoxy nanocomposites is essential. While several studies report on the quasi-static mechanical behavior of silica/epoxy nanocomposites, few results are available regarding their high strain rate behavior. Miao et al. [32] showed that adding silica nanoparticles of size 20 nm and 10% weight content to epoxy only marginally improved its compressive yield strength at strain rates up to 5000 s^−1^. Additionally, a significant strain rate sensitivity was reported, where the yield strength increased with increasing the strain rate. Tian et al. [33] reported that the addition of 30 nm silica nanoparticles with 10% weight content increased the compressive modulus and yield strength of epoxy resin with increasing strain rates up to 3000 s^−1^. However, the improvement of the compressive performance was more pronounced in the low strain rate regime compared to the high strain rate regime. Contrary to these findings, Guo et al. [34] showed that the improvement in the compressive strength by the addition of 90 nm silica nanoparticles up to 7% weight content was more pronounced at high strain rates up to 10^4^ s^−1^, whereas no improvement could be observed at low strain rates. Ma et al. [35] showed that for epoxy filled with silica nanoparticles of size 50 nm and up to 15% weight content, the compressive failure strength increased at strain rates up to 200 s^−1^ and higher silica contents. The compressive stiffness, however, showed a reduction at higher silica weight contents. Yohanes [36] found that the addition 17 nm silica nanoparticles increased the dynamic stiffness of the epoxy at high strain rates, regardless of the weight content of the particles. However, when mixed with 34 µm silica particles, the dynamic stiffness is significantly reduced.

The previous literature overview clearly indicates that the experimental data are contradicting, and no clear trends can be established regarding the effect of strain rate and silica particles size and content on the mechanical behavior of epoxy resins. Despite the contradictions, part of the data still suggests that a lower weight content of silica particles, combined with a submicron size scale, has the potential to improve the mechanical properties of the epoxy resin without compromising its thermal or physical properties. This can be achieved by silica nanoparticle sizes of 300 nm up to 1 µm. However, to the best of the authors’ knowledge, no data are available in the literature regarding the effect of strain rate on the compressive properties of epoxy resins filled with silica nanoparticles within this specific size range.

The aim of the present paper is to study the effect of strain rate and silica filler content on the compressive behavior of epoxy resin. The aeronautical grade RTM6 was considered, as it is suitable for low volume aircraft structures made by the resin transfer molding technique. Silica nanoparticles of sizes 300 nm and 800 nm with different surface functionalization conditions and weight percentages of 0.1%, 1% and 5% (5% wt. only for non-functionalized particles) and were investigated. High strain rate compression experiments were performed using a split Hopkinson pressure bar test (SHBT) setup. Reference quasi-static experiments were also performed in order to study the compressive behavior at a wide range of strain rates. In order to obtain accurate values of the multiaxial strain components, full-field strain measurements were performed using stereo digital image correlation techniques (DIC). The effect of strain rate on the compressive stiffness, Poisson’s ratio and peak yield strength is discussed. In addition, the effect of the weight content, the size, and the surface functionalization conditions of the silica nanoparticles on the compressive behavior of epoxy nanocomposite at different strain rates is presented.

## 2. Materials and Methods

### 2.1. Matrix Material

The epoxy resin used in this study was the aeronautical grade RTM6, supplied by Hexcel Composites (Duxford, Cambrige, UK). It was made up of tetra-functional epoxy resin tetraglycidyl methylene dianiline (TGMDA) and two hardeners, namely 4,4′-methylenebis (2,6-diethylaniline) and 4,4′-methylenebis (2-isopropyl-6-methylaniline). The equivalent weight of the epoxy after mixing with the hardeners was 116 g/eq and the viscosity was 33 mPa·s at 120 °C. For the synthesis of the silica nanoparticles, tetraethyl orthosilicate (TEOS), 3-aminopropyl triethoxysilane (APTES) and other solvents supplied by Sigma-Aldrich (St. Louis, MO, USA) were used. All the chemicals were used as-received.

### 2.2. Nanoparticles Synthesis and Nanocomposite Preparation

**Figure 1:**
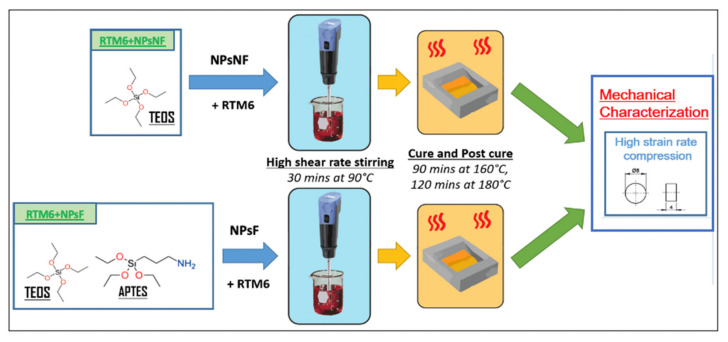
Manufacturing procedures of the silica/epoxy nanocomposites.

**Figure 2:**
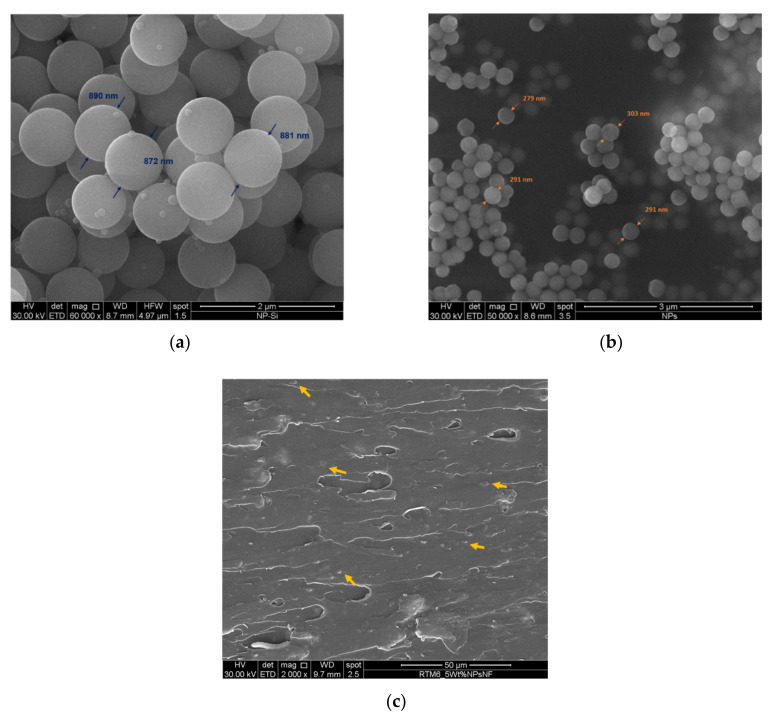
SEM images of the synthesized silica nanoparticles: (**a**) non-functionalized, (**b**) functionalized and (**c**) non-functionalized nanoparticles (indicated by arrows) at 5% weight content in the RTM6 epoxy resin.

Non-functionalized silica nanoparticles (NPsNF) were prepared using the Stöber method [37] with TEOS as precursors. TEOS (19.6 mL) was added drop by drop, while stirring to an alcoholic solution containing 50 mL of ethanol, 18 mL of water and 6.3 mL of ammonia. The mixture was then heated under reflux at 78 °C for 68 min. The solution was filtered and washed with deionized water, then dried in a vacuum oven (SALVIS VC20, Germany) overnight at 90 °C. The same procedure was employed for the synthesis of the functionalized silica nanoparticles (NPsF), however, an equimolar mixture of 9.8 mL TEOS and 10.3 mL APTES was employed instead of only TEOS [38]. Figure 1 shows a schematic illustration of the manufacturing process of the nanocomposites. The average diameter of the non-functionalized silica nanoparticles was 880 nm, whereas the average diameter of the functionalized silica nanoparticles was 300 nm, as depicted from the SEM images of the prepared nanoparticles (see Figure 2a,b). The Scanning Electron Microscopy (SEM) images were analyzed by ImageJ software (version 1.53m) and at least 15 particles were used to measure the average particle diameters. The reason for the size difference could be attributed to the functionality of the APTES precursor which is characterized by only three reactive functional groups (O-CH_2_CH_3_) compared to TEOS which has four reactive functional groups. The reduced functionality of APTES limits the nanoparticle growth, thus, explaining the smaller dimensions of the functionalized silica nanoparticles. Table 1 lists the composition of the manufactured nanocomposites.

The RTM6 resin was prepared by first degassing the resin at 90 °C for 30 min in a vacuum oven, then the hardener was added and carefully mixed, according to the specified mixing ratio by the manufacturer. Different weight contents of nanoparticles were mixed in the resin using a high shear rate mixer (T25 digital ULTRA-TURRAX, from IKA, Staufen, Germany) to ensure a uniform dispersion, as depicted in Figure 2c. The weight contents of the non-functionalized silica nanoparticles were 0.1%, 1% and 5%, while the weight contents of the functionalized silica nanoparticles were 0.1% and 1%. The unreacted mixes of the resin and the silica nanoparticles were molded into long, hollow metallic cylinders which were coated with a release agent (FREKOTE 70 manufactured by Henckel, Rocky Hill, CT, USA) to facilitate the extraction of the samples. The resins were cured in an oven at 160 °C for 90 min, followed by a post-curing stage of 2 h at 180 °C, and left to cool to room temperature in the oven for 24 h.

The fully cured cylindrical rods of both neat and filled resins were finally cut into small cylindrical samples, having a diameter of 8 mm and a height of 4 mm. The selected height-to-diameter ratio of 0.5 helps to reduce the effects of interfacial friction during compression, which can give rise to significant sample barreling [39]. To eliminate any discrepancies related to the sample geometry, the same sample geometry and testing boundary conditions were used for both reference quasi-static and high strain rate tests.

### 2.3. Dynamic Mechanical Analysis

Dynamic mechanical analysis (DMA) was performed by using the DMA Q800 system manufactured by TA Instruments. Double cantilever testing mode was employed on samples of neat and filled epoxy resins having nominal dimension of 60 mm × 12 mm × 2.5 mm. Samples were tested at an amplitude of 60 μm, a frequency of 1 Hz and a heating rate of 3 °C/min. Three tests for each silica nanoparticle concentrations were performed.

### 2.4. Quasi-Static Testing

Reference quasi-static experiments were carried out on the cylindrical samples using an Instron 5569 universal testing machine (supplied by Instron, Boechout, Belgium). The height of each sample was 4 mm and the diameter was 8 mm. Quasi-static compression tests were performed at speeds of 0.2, 2 and 20 mm/min, aiming at strain rates of 0.001, 0.01 and 0.1 s^−1^, respectively, in the samples. Samples were placed between two flat ended steel bars, whose loading interfaces were polished to a mirror finish and lubricated with a PTFE lubricant to reduce friction. To eliminate any discrepancy related to sample geometry or boundary conditions, the same sample geometry and boundary conditions used in the quasi-static testing were used in the high strain rate testing.

**Figure 3:**
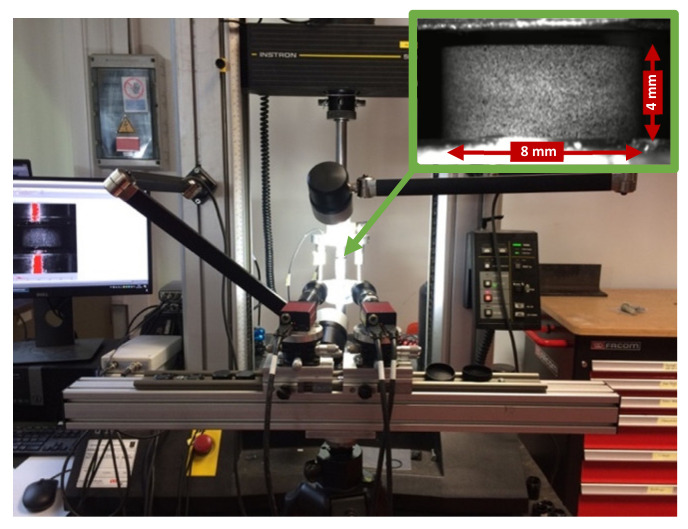
Quasi-static compression setup with a detail of the speckled sample (top right).

Global displacements and strains were measured using 3 linear variable displacement transducers LVDTs (supplied by RDP Group, Le Spijkenisse, The Netherlands) fixed on the bars close to the sample. The measurements obtained from the LVDTs were later corrected for the bar compliance during compression. Local full-field displacements and strains were measured using a low speed 3D DIC setup. For this purpose, the lateral surfaces of the compression samples were painted with a black-on-white speckle pattern before testing. The deformation of the samples, from their speckle pattern, was recorded by two machine vision cameras (stingray 504b by Allied Vision, Stadtroda, Germany) positioned under a stereo angle of 18.85° (value obtained after a calibration procedure). Images were recorded at a resolution of 2452 × 2056 pixels and a rate of 1 images/s for the testing speeds of 0.2 and 2 mm/min, and 3 image/s for the testing speed 20 mm/min. Figure 3 shows the quasi-static setup used.

### 2.5. High Strain Rate Testing

**Figure 4:**
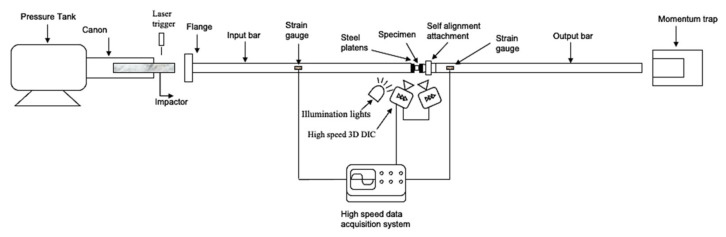
Schematic of the SHPB setup.

The high strain rate compression experiments were performed using the split Hopkinson pressure bar (SHPB) facility available at MST-DyMaLab at Ghent University. The details of the setup were explained in previous work [11]. Figure 4 shows a schematic of the SHPB setup used. The cylindrical sample was placed between two long, aluminum bars, called input and output bars. The dynamic incident compressive loading wave was generated by accelerating the impactor towards a flange at the end of the input bar.

The interaction of the compressive incident loading wave with the sample resulted in the wave being partly reflected back to the input bar, and partly transmitted to the output bar. Using strain gauges attached to both bars at well-chosen locations, the strain histories corresponding with the reflected wave $\varepsilon_{r}\left(t\right)$ and transmitted wave $\varepsilon_{t}\left(t\right)$ were measured. A time shifting is applied to the measured waves to shift them from the strain gauge locations on the bars to the interfaces of the bars and sample [40]. The loading time of the incident wave was ~1.2 ms, which is long enough for the samples to reach yielding. The chosen sample geometry and small dimensions guaranteed that, from the early stage of deformation, the sample reached a state of quasi-static force equilibrium. Additionally, inertia effects were negligible and sufficiently high strain rates were achieved in the dynamic tests [41,42]. In that case, using the one dimensional wave propagation analysis developed by Kolsky [42], the time histories of the average axial strain rate ${\dot{\varepsilon }}^{Hop}$, strain $\varepsilon^{Hop}$ and stress $\sigma^{Hop}$ in the sample can be calculated as follows:

(1) $${\dot{\varepsilon }}^{Hop}=-2\frac{C_{o}}{H_{s}}\varepsilon_{r}\left(t\right)$$

(2) $$\varepsilon^{Hop}=-2\frac{C_{o}}{H_{s}}\int_{0}^{t}\varepsilon_{r}\left(t\right)dt$$

(3) $$\sigma^{Hop}=E_{b}\frac{A_{b}}{A_{s}}\varepsilon_{t}\left(t\right)$$

where $C_{0}$ is the elastic wave speed in the bar material; $H_{s}$ is the height of the sample; $E_{b}$ is the elastic modulus of the bar material; and $A_{b}$ and $A_{s}$ are the cross section areas of the bar and the sample, respectively. Assuming conservation of volume, the axial compressive true stress $\sigma_{t}^{Hop}$ and axial true strains $\varepsilon_{t}^{Hop}$ based on the Hopkinson analysis, i.e., Equations (2) and (3), can be calculated using the following relations:

(4) $$\sigma_{t}^{Hop}=\sigma^{Hop}\left(1+\varepsilon^{Hop}\right)$$

(5) $$\varepsilon_{t}^{Hop}=\ln\left(1+\varepsilon^{Hop}\right)$$

**Figure 5:**
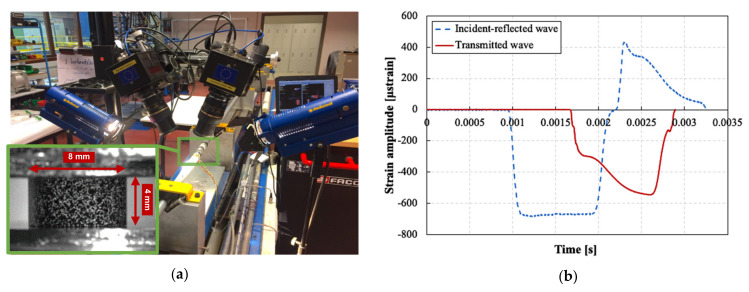
(**a**) High-speed 3D DIC setup used, with detail of the speckled sample (bottom left). (**b**) An example of the incident, reflected and transmitted waves recorded by strain gauges on the Hopkinson bars during a dynamic compression experiment.

A high-speed 3D DIC technique was used to measure the local strains and strain rates on the surface of the sample. Similar to the quasi-static DIC setup, the lateral surfaces of the samples were painted with a thin black-on-white speckle pattern prior to testing. The deformation of the speckle pattern was recorded using two high-speed cameras (Photron Mini AX200) positioned at a stereo angle of 26.36° (value obtained after a calibration procedure). Images were recorded at a resolution of 384 × 265 pixels^2^ and a rate of 54,000 images/s. Figure 5a shows the high-speed stereo DIC setup used.

Figure 5b shows an example of the incident, the reflected and the transmitted waves recorded in one of the dynamic compression experiments. To impose different strain rates to the sample, three impactor velocities were used: 8, 11 and 14 m/s. Note that some factors, such as sample indentation into the bars and the contact conditions between the bars and sample interfaces, could influence the accuracy of the split Hopkinson bar technique, especially in small strains measurement range [43]. To eliminate these sources of error, a self-alignment attachment was fixed to the output bar, as shown in Figure 4. This attachment was especially designed to reduce the impedance mismatch effects on the propagating waves, as evident in the strain signals of Figure 5b. Moreover, the self-alignment attachment ensured perfect contact between the specimen and the bar interfaces, even if the sample interfaces are slightly tilted. Additionally, two thin steel plates were attached to the loading interfaces of the sample to eliminate any indentation into the bars. Furthermore, the loading interfaces of the sample were lubricated with a PTFE based lubricant to minimize the interfacial friction.

### 2.6. DIC Data Reduction and Processing Parameters

The MatchID commercial digital image correlation software (supplied by MatchID, Ghent, Belgium) was used to analyze and process the images of the deformed samples during the tests. Table 2 shows the processing parameters used for both quasi-static and dynamic tests. These parameters allowed to achieve a strain resolution of ~155 microstrains for quasi-static tests and ~400 microstrains for high strain rate tests. At each moment during the quasi-static and dynamic tests, the average full field in-plane strains and out-of-plane displacements were extracted from an area of 3.5 mm × 3.5 mm at the center of the sample. The axial engineering and true strains $\varepsilon^{DIC}$ and $\varepsilon_{t}^{DIC}$ were calculated based on the reference Biot and Hencky strain conventions, respectively. In order to increase the accuracy of the axial compressive true stress, its value was also calculated based on the transverse component of the strain $\varepsilon_{hoop}^{DIC}$ (i.e., hoop strain) obtained using stereo DIC measurements. Therefore, it was possible to calculate the axial compressive true stress based on the instantaneous cross section, without the need to assume volume conservation as was the case for Equation (4). For this purpose, the following relation can be used:

(6) $$\sigma_{t}^{DIC}=\frac{F}{A}=\frac{F}{\pi r^{2}}=\frac{F}{\pi r_{0}^{2}{\left(1+\varepsilon_{hoop}^{DIC}\right)}^{2}}=\frac{\sigma^{Hop}}{{\left(1+\varepsilon_{hoop}^{DIC}\right)}^{2}}$$

## 3. Results and Discussion

### 3.1. Dynamic Mechanical Analysis

**Figure 6:**
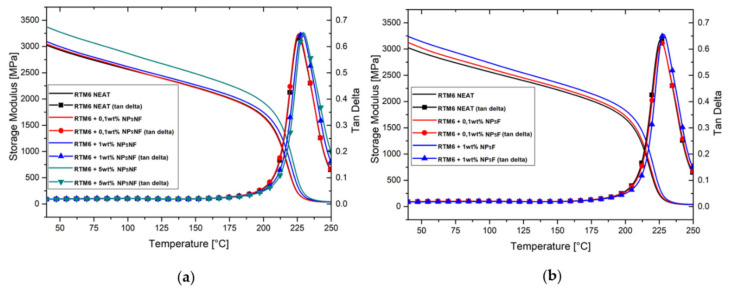
DMA curves of the silica/RTM6 epoxy nanocomposites compared to the neat RTM6 epoxy: (**a**) NPsNF and (**b**) NPsF filled nanocomposites.

The dynamic mechanical properties of silica-based nanocomposites were examined within the temperature range of 40 °C to 250 °C by DMA analysis. Figure 6 shows both the storage modulus and loss factor (tan delta) as a function of temperature for both nanocomposite types. It can be seen that the addition of the silica nanofillers improved the storage modulus of the RTM6 epoxy resin. The largest increase in the storage modulus, of ~11.6%, was associated with the addition of 5 wt% of non-functionalized silica nanoparticles to the neat resin. It is well known in the literature [44] that the addition of silica particles of micro and nanoscale sizes increase the storage modulus of the hosting matrix, both in the glassy and rubbery regions, due to the reduction in the free volume of the matrix. Moreover, the addition of the silica nanofillers also slightly increased the glass transition temperature of the RTM6 epoxy resin, as evident from the temperatures corresponding to the peak of the tan delta curves. This behavior is attributable to the higher surface area of the smaller nanoparticles. In fact, the greater surface of interaction between filler and matrix limits the thermal movements of the polymeric chains, causing the increase of the glass transition temperature [31]. Table 3 shows the results of the DMA.

### 3.2. Compressive Stress-Strain Response of RTM6 Epoxy Nanocomposites at Different Strain Rates

**Figure 7:**
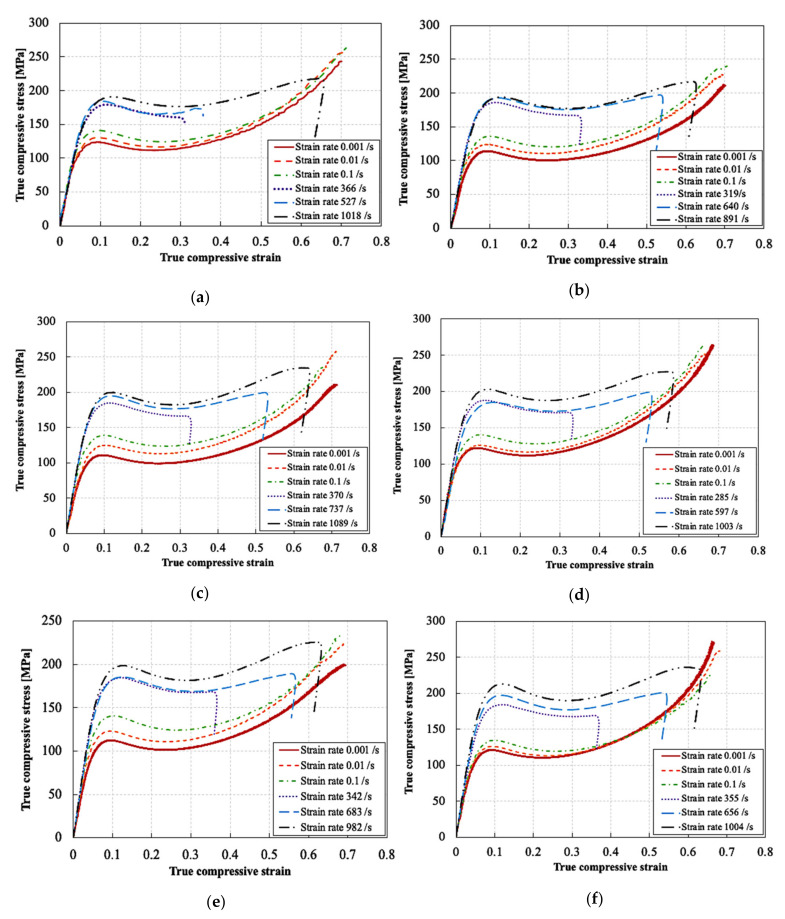
Representative compressive true stress-true strain curves at different strain rates for the RTM6 epoxy/silica nanocomposites: (**a**) unfilled neat resin, (**b**) non-functionalized 0.1%, (**c**) non-functionalized 1%, (**d**) non-functionalized 5%, (**e**) functionalized 0.1% and (**f**) functionalized 1%.

**Figure 8:**
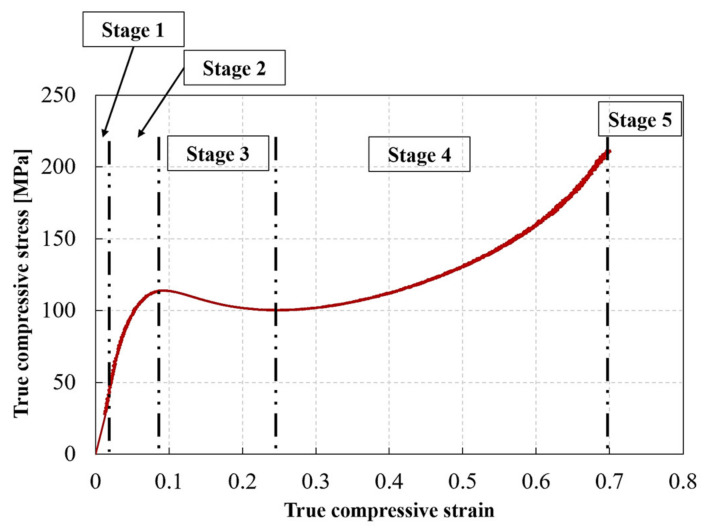
Stages of deformation of a silica nanoparticle sample (0.1% NPsNF) in compression.

Figure 7 shows representative true stress–true strain curves for the RTM6 epoxy nanocomposites at different strain rates and particle weight contents. At least three experiments were performed for each testing condition. Note that the speckle pattern could not follow the deformation of the samples beyond ~30% true strain. Therefore, the quasi-static and high strain rate curves in Figure 7 are based on the LVDT and classical Hopkinson analysis (Equations (4) and (5)), respectively, as this information—unlike the DIC data—is available until fracture or unloading. For all the dynamic compression tests, the stresses at both bar interfaces were calculated using the 1D wave propagation theory, to confirm the achievement of quasi-static equilibrium. As equilibrium was established from the early stages of deformation, and the errors in the small deformation range were experimentally reduced, it was possible to calculate the elastic modulus and Poisson’s ratio at high strain rates. Accurate values were obtained using the DIC strains. Moreover, the evolution of the true strain rate (i.e., true strain—time curve obtained by DIC) revealed a bilinear behavior, and consequently two stages with a different, yet relatively constant strain rate, separated with a transition point at ~0.07 true strain. Therefore, the strains rates corresponding to the elastic constants of the material were calculated in the first stage, i.e., from strain values of 0 to 0.07, while the strain rates corresponding to the yielding of the material were calculated in the second stage, i.e., from strain values 0.07 to 0.3. The strain rates indicated in Figure 7 correspond to the strain rate in the second stage. It can be seen that the compressive behavior of all tested epoxies, i.e., neat and filled, is highly strain rate sensitive. The true stress–true strain response for all materials follows 5 distinct stages, as depicted in Figure 8: (1) an initial, linear stage corresponding to the material’s viscoelastic behavior; (2) a nonlinear stage corresponding to the yielding of the material [45], which reaches a maximum value at the peak yield point [45,46]; (3) a strain softening stage following the yielding and (4) further strain hardening; and (5) fracture for the quasi-static strain rates, or unloading for the high strain rates. Note that all the statically tested samples were loaded until fracture, whereas all the dynamically tested samples were not fractured at the end of loading and spring back during unloading was observed. Therefore, the strains at unloading cannot be considered a material property. All tested epoxies, filled and unfilled, showed an increase in strength with increasing strain rates. Both Gerlach et al. [41] and Morelle et al. [45] reported similar trends for RTM6 neat resin. Table 4 summarizes the results of all compression tests. The elastic modulus was calculated as the slope of the true stress–true strain curve between in the true axial strain range of 0 to 0.02, based on DIC strain measurements and Equation (6). The Poisson’s ratio was calculated as the slope of the hoop strain–axial strain curve in the same strain range, based on DIC strain measurements. The peak yield strength was considered as the strength at the peak yield point [45,46], and was obtained based on the instantaneous cross section area, i.e., using Equation (6).

### 3.3. Effect of Strain Rate and Weight Content on the Elastic Modulus and Poisson’s Ratio of the Silica Nanoparticles Filled RTM6 Epoxy Resin

**Figure 9:**
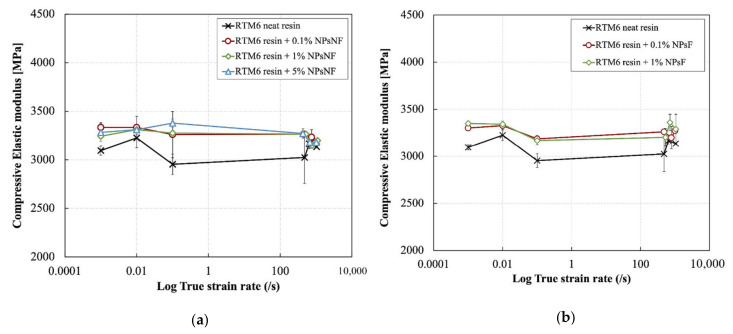
Effect of strain rate on the elastic modulus of the silica nanoparticle-filled epoxy at different particle weight contents and functionalization conditions: (**a**) non-functionalized and (**b**) functionalized.

**Figure 10:**
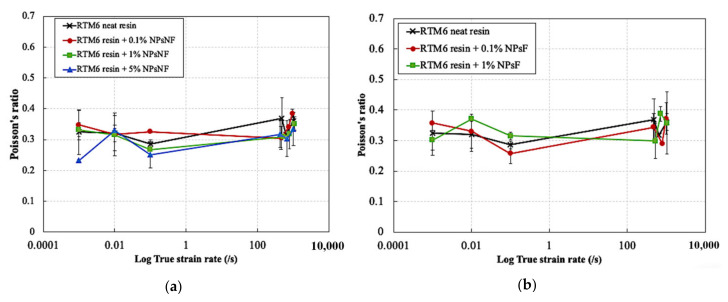
Effect of strain rate on the Poisson’s ratio of the silica nanoparticle-filled epoxy at different particle weight contents and functionalization conditions: (**a**) non-functionalized and (**b**) functionalized.

Figure 9 and Figure 10 show the effect of strain rate on the elastic modulus and the Poisson’s ratio of silica nanoparticle-filled epoxy resins at different particle weigh contents and functionalization conditions. The elastic modulus was slightly improved by the addition of the silica nanoparticles with different weight percentages of the particles across all strain rates. The elastic modulus of the neat resin was increased from ~3100 MPa to ~3300 MPa by the addition of silica nanoparticles. This corresponds to an increase of ~6.4%. Moreover, the elastic modulus was hardly affected by strain rate. Increasing the weight content of both particle types up to 5% also did not have a significant effect on the elastic modulus across all strain rates.

The Poisson’s ratio also is independent of strain rate for all materials. The average value of the Poisson’s ratio for the neat and silica nanoparticle filled epoxies was ~0.32. The slight increase in the elastic modulus by the addition of the silica nanoparticles is due to the transfer of forces from the matrix to the higher stiffness particles. As seen earlier in Section 3.1, the epoxy resin showed a predominantly elastic behavior rather than a viscoelastic behavior at the early stages of loading based on the DMA results. Consequently, the resin material is expected to show a strain rate independent response at small strains due to the lack of contribution of the damping component in the deformation behavior at high strain rates.

### 3.4. Effect of Strain Rate and Weight Content on the Peak Yield Strength of the Silica Nanoparticle-Filled RTM6 Epoxy Resin

**Figure 11:**
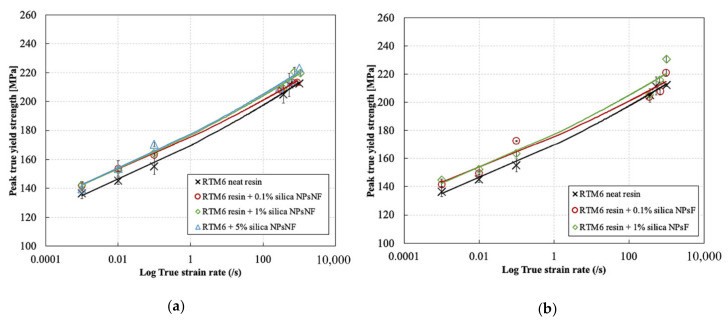
Effect of strain rate on the compressive true peak yield strength for the silica nanoparticle-filled epoxy at different particle weight contents and functionalization conditions: (**a**) non-functionalized and (**b**) functionalized.

Figure 11 shows the effect of strain rate on the true peak yield strength for functionalized and non-functionalized silica nanoparticle filled epoxy resins. The addition of the silica nanoparticles with different weight contents improved the true peak yield strength at different strain rates compared to the neat resin. Hardly any change was observed in the trend of the true peak yield strength with increasing strain rate as a result of the surface functionalization conditions of the silica nanoparticles. Compared to the neat resin at low strain rates, the peak yield strength of the 0.1% silica content increased from 116.4 MPa to 122.42 MPa at strain rate of 0.0008 s^−1^ and from 135.19 MPa to 143.18 MPa at strain rate of 0.08 s^−1^. This corresponds to percentage increase of 5.2% and 5.9%, respectively. This increase was almost the same regardless of the filler content in the low strain rate range up to 0.08 s^−1^.

Moreover, it can be seen that the improvement of the peak yield strength was much more significant in the low strain rate range compared to the high strain rate range. Indeed, compared to the neat resin, the peak yield strength of the 0.1% silica nanoparticle content increased from 184.84 MPa to 187.95 MPa at strain rate of 277.5 s^−1^ and 303.7 s^−1^, respectively, and from 192.3 MPa to 193.39 MPa at strain rate of 1019.65 s^−1^ and 904.1 s^−1^. This corresponds to percentage increases of 1.6% and 0.56%, respectively, which are much lower compared to the percentage increases in the low strain rate range. The most significant improvement of the true peak yield strength was observed at silica weight contents of 1% and 5%, especially at low strain rates. Similar results were also reported by Tian et al. [33] and Miao et al. [32], but for a different epoxy formulation and a different size of nanoparticles. The increase in the true peak yield strength of the epoxy/silica nanocomposite is attributed mainly to the viscoelastic nature of the resin. However, further research is still required to understand why the improvement of the yield strength is reduced at high strain rates compared to low strain rates. At low strain rates, the viscoelastic resin has enough time to deform. This allows the transfer of the forces from the matrix to the higher strength and higher stiffness silica nanoparticles, which further increase the yield strength. However, at high strain rates, the viscoelastic resin does not have enough time to fully deform due to the reduced molecular mobility of the polymer chains, as demonstrated by Chen et al. [47]. The reduced molecular mobility at high strain rates could reduce the interaction between the resin and the silica nanoparticles [48]. This behavior was also observed for carbon nanotube fillers, as reported by Del Rio et al. [9].

Another contributing factor is the adiabatic heating at high strain rates, which cannot be neglected. Indeed, Pan et al. [49] reported that the temperature rise in epoxy samples due to adiabatic heating at high strain rates can reach up to 90 °C. Furthermore, it was reported by Del-Rio et al. [50] that an increase of 40 °C can reduce the yield strength of epoxy by 23% at high strain rates. Note that Miao et al. [32] suggested that the strain softening of the epoxy matrix is the main contributing factor of the reduction in the yield strength at higher strain rates regardless of the nanoparticle weight percentage added. However, this conclusion was based on a simple model which was validated only for silica nanoparticle content of 10% and cannot be directly extended to other highly crosslinked epoxy resins and other weight percentages of silica nanoparticles.

The increasing trend of the true peak yield strength of the silica filled epoxies with increasing strain rate can be described by a least square power fit relation as follows (R^2^ values are greater than 0.9):

(7) $$\sigma_{t}^{Peak\;yield}=C{\dot{\varepsilon }}^{d}$$

where $\sigma_{t}^{Peak\;yield}$ is the true peak yield strength in compression, *C* is the compressive strength coefficient and *d* is the strain rate sensitivity exponent. For the non-functionalized silica filled epoxy resin, the compressive strength coefficients for the 0.1%, 1% and 5% weight contents were 154.85 MPa, 156.13 MPa and 157.04 MPa respectively, while the strain rate sensitivity exponents for the same consecutive weight contents were 0.0335, 0.0353 and 0.0354, respectively. For the functionalized silica-filled epoxy resin, the compressive strength coefficients for the 0.1% and 1%, weight contents were 155.09 MPa and 156.96 MPa, respectively, while the strain rate sensitivity exponents for the same consecutive weight contents were 0.0331 and 0.0355, respectively.

### 3.5. Effect of the Silica Nanoparticles Size and Surface Functionalization of on the Elastic Modulus, Poisson’s Ratio and Peak Yield Strength of RTM6 Epoxy Nanocomposite

**Figure 12:**
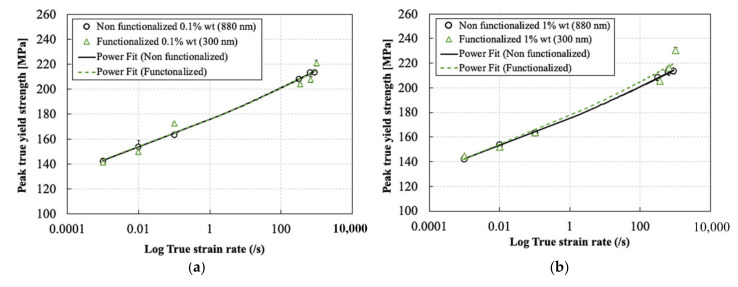
Effect of silica nanoparticles size and surface functionalization on the compressive true peak yield strength: (**a**) 0.1% and (**b**) 1%.

**Figure 13:**
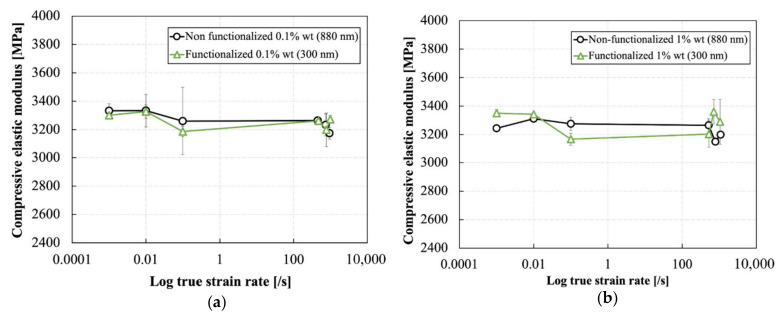
Effect of silica nanoparticles size and surface functionalization on the compressive elastic modulus for the silica nanoparticle-filled epoxy at different particle weight contents: (**a**) 0.1% and (**b**) 1%.

**Figure 14:**
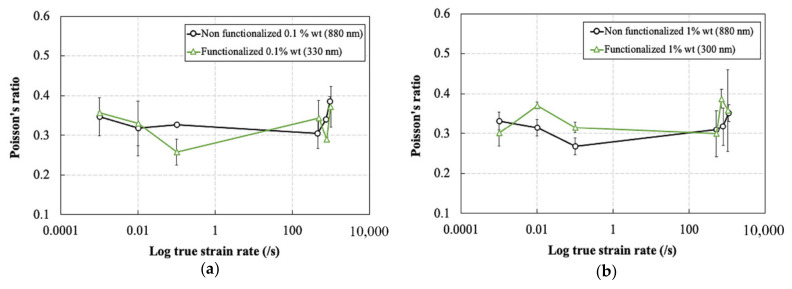
Effect of silica nanoparticles size and surface functionalization on the Poisson’s ratio for the silica nanoparticle: (**a**) 0.1% and (**b**) 1%.

Despite the different surface functionalization conditions of the silica nanoparticles used in this study, a rough estimate of the effect of the different sizes of these nanoparticles on the compressive behavior of the epoxy resin can still be studied. Figure 12, Figure 13 and Figure 14 show the effect of the silica nanoparticles size and surface functionalization conditions on the peak true yield strength, elastic modulus, and Poisson’s ratio, respectively, for weight percentages of 0.1% and 1% at different strain rates. It can be seen that for a silica nanoparticle content of 0.1%, the size of the particles and the surface functionalization conditions did not have a significant effect on the true peak yield strength and the elastic modulus all strain rates. Whereas for a silica nanoparticle content of 1%, a very slight increase in the true peak yield strength can be seen at the high strain rate range as a result of reducing the particle size from 880 nm to 300 nm and the functionalization of the particle surface. The size and the surface functionalization of the nanoparticles also did not show a significant effect on the elastic modulus and the Poisson’s ratio at different strain rates for both filler contents. Similar results were reported by Dittanet et al. [29] for a similar epoxy system at quasi-static strain rates and silica nanoparticle size range from 23 nm to 170 nm. Here, again, further research is required to understand why the change of the silica nanoparticle sizes in the range 300 nm to 880 nm does not significantly affect the compressive properties of the epoxy resin, particularly at high strain rate. As explained earlier, the combined effect of viscoelasticity and adiabatic heating could be the main contributing factors in that case.

## 4. Conclusions

Several experiments were performed to study the effect of strain rate and filler content on the compressive behavior of several epoxy-based nanocomposites. The aeronautical grade RTM6 epoxy was filled with silica nanoparticles of sizes 300 nm and 880 nm and different surface functionalization conditions. Three weight percentages were considered for the fillers: 0.1%, 1% and 5% (5% wt. only for non-functionalized particles). Quasi-static and high strain rate compression experiments were performed using a universal testing machine and a SHPB setup, respectively, to cover a strain rate range from 0.001 s^−1^ up to 1100 s^−1^. Local displacements and strains in the sample were measured using the 3D digital image correlation technique. The effect of the strain rate, the size and the weight percentage of the silica nanoparticles on the elastic modulus, the Poisson’s ratio and the true peak yield strength of the tested materials were discussed. Considering the tested materials, manufacturing techniques used, testing equipment and results, the following can be concluded:

1. The tested RTM6 neat and nanoparticle filled resins were all strain rate-sensitive in compression. All materials showed an increase in strength with increasing strain rates for all the weight percentages and sizes of the fillers.
2. The elastic modulus and Poisson’s ratio of the tested epoxy nanocomposites were independent of the strain rate and showed a nearly constant behavior at different strain rates for all weight percentages and sizes of the particles. However, the true peak yield strength showed an increase with increasing strain rates for all weight percentages and sizes of the particles used.
3. The addition of silica nanoparticles to the RTM6 epoxy resin generally improved both its elastic modulus and its peak yield strength at different strain rates for all the weight percentages of the particles. Increasing the weight percentage of both types the silica nanoparticles from 0.1% to 5% did not yield any improvement in the elastic modulus and the Poisson’s ratio but led to a slight increase in the peak yield strength. Additionally, it was found that the improvement in the peak yield strength due to the addition of silica nanoparticles was more prominent in the quasi-static strain rate regime compared to the high strain rate regime.
4. The dynamic mechanical analysis showed an increase in the storage modulus and a marginal increase in the glass transition temperature of the resin by the addition of silica nanoparticles of different weight percentages.
5. The sizes of the silica nanoparticles used (300 nm and 880 nm) did not significantly affect the compressive properties of the RTM6 epoxy resin, regardless of the weight percentages of the particles.

## Figures and Tables

**Table 1:** Composition of the manufactured nanocomposites.

Sample	Matrix	Filler	Filler Content [%]
RTM6 neat resin	RTM6	-	0
RTM6 + 0.1 wt% NPsNF	RTM6	NPsNF	0.1
RTM6 + 1 wt% NPsNF	RTM6	NPsNF	1
RTM6 + 5 wt% NPsNF	RTM6	NPsNF	5
RTM6 + 0.1 wt% NPsF	RTM6	NPsF	0.1
RTM6 + 1 wt% NPsF	RTM6	NPsF	1

**Table 2:** Processing parameters for DIC.

Parameter	Value
Correlation criterion	Zero normalized sum of square differences (ZNSSD)
Interpolation order	Bi-cubic spline
Shape function	Affine
Subset size (pixels x pixels)	55 × 55 (quasi-static) and 21 × 21 (high strain rate)
Step size (pixels)	10
Strain window	15

**Table 3:** Dynamical mechanical analysis results.

Sample Type	Storage Modulus at 40 °C (MPa)		Storage Modulus at 250 °C (MPa)		Glass Transition Temperature (°C)
	Mean	Std. Dev	Mean	Std. Dev	
RTM6 neat resin	3023	±23	38.4	±2.1	226.6 ± 0.2
RTM6 + 0.1 wt% NPsNF	3047	±43	38.8	±1.8	225.7 ± 0.3
RTM6 + 1 wt% NPsNF	3093	±17	39.4	±1.7	229.0 ± 0.2
RTM6 + 5 wt% NPsNF	3375	±33	44.5	±2.4	229.4 ± 0.4
RTM6 + 0.1 wt% NPsF	3123	±56	39.9	±6.2	226.0 ± 0.3
RTM6 + 1 wt% NPsF	3243	±31	41.0	±3.1	228.3 ± 0.4

**Table 4:** Summary of the results of the neat RTM6 and RMT6/ silica epoxy nanocomposite (functionalized and non-functionalized) during compression at different strain rates.

Material Condition	Achieved True Strain Rates for Elastic Modulus and Poisson’s Ratio (s^−1^)	Elastic Modulus (MPa)		Poisson’s Ratio		Achieved True Strain Rates for Peak Yield Strength (s^−1^)	True Peak Yield Strength (MPa)	
		Mean	Std. Dev.	Mean	Std. Dev.		Mean	Std. Dev.
RTM6 neat resin	0.001	3095.653	46.804	0.3243	0.1256	0.001	136.403	6.2101
	0.01	3225.250	100.59	0.3208	0.0985	0.01	145.318	4.2153
	0.1	2953.969	105.521	0.2861	0.0178	0.1	155.196	7.8117
	478.638	3024.542	267.920	0.3683	0.09698	365.760	204.840	8.2448
	638.877	3166.459	52.097	0.3174	0.0017	527.334	211.603	11.305
	1034.972	3135.218	9.455	0.3608	0.0375	1017.751	212.295	1.774
RTM6 + 0.1% NPsNF	0.001	3332.913	69.506	0.3471	0.0675	0.001	142.063	3.574
	0.01	3333.440	162.954	0.3175	0.0978	0.01	153.812	7.4526
	0.1	3259.936	336.447	0.3259	0.0045	0.1	163.187	1.1321
	454.339	3264.105	1.148	0.3046	0.0531	318.821	207.955	0.994
	750.6	3233.544	109.158	0.3398	0.0073	640.054	213.176	0.717
	946.35	3174.352	58.153	0.3846	0.0189	891	213.292	0.9457
RTM6 + 1% NPsNF	0.001	3243.419	5.8576	0.3318	0.0325	0.001	146.242	0.873
	0.01	3310.858	26.257	0.3147	0.2677	0.01	152.376	0.0631
	0.1	3275.084	65.296	0.2677	0.0300	0.1	164.471	0.1899
	520.731	3264.376	79.535	0.3090	0.0013	369.592	208.867	3.8367
	786.096	3150.037	28.658	0.3172	0.0675	737.255	220.455	5.8581
	1093.333	3198.981	44.3911	0.3516	0.0306	1089.456	219.829	3.6738
RTM6 + 5% NPsNF	0.001	3282.311	53.296	0.2324	0.0038	0.001	139.690	6.1066
	0.01	3310.217	130.112	0.3299	0.0240	0.01	153.717	1.6009
	0.1	3378.446	73.952	0.2516	0.0597	0.1	170.631	0.4492
	431.035	3270.771	70.508	0.3174	0.0607	285.279	209.522	2.3349
	670.14	3180.850	83.435	0.3031	0.0809	596.957	214.067	0.5062
	1015.2	3179.854	107.427	0.3341	0.0766	1003.242	222.986	2.6408
RTM6 + 0.1% NPsF	0.001	3301.023	3.642	0.3573	0.0028	0.001	141.454	2.6183
	0.01	3327.148	66.729	0.3300	0.0792	0.01	149.692	1.6139
	0.1	3186.456	50.550	0.2572	0.0465	0.1	172.510	0.6441
	476.222	3261.809	25.714	0.3439	0.0621	342.424	203.886	1.0950
	782.692	3198.150	205.811	0.2900	0.0068	683.038	207.785	3.3535
	991.71	3273.225	45.430	0.3723	0.072	982.414	220.873	3.3542
RTM6 + 1% NPsF	0.001	3349.470	36.207	0.3013	0.0457	0.001	144.687	0.9358
	0.01	3342.377	10.823	0.3707	0.0112	0.01	152.181	3.4609
	0.1	3166.964	60.340	0.3152	0.0189	0.1	163.443	3.0203
	524.656	3202.538	132.026	0.2990	0.0818	355.420	205.585	1.7706
	711.72	3360.668	122.428	0.3875	0.0334	656.1	215.882	2.0841
	1048.6	3290.045	222.304	0.3576	0.1438	1004.4	230.579	3.1928

## Data Availability

The data presented in this study are available on request from the corresponding author. The data are not publicly available due to confidentiality.
